# Remarks on a Class of Pseudo-Inflammatory Affections

**Published:** 1829-10-01

**Authors:** 


### Remarks on a peculiar Class of Diseases, resembling Inflammation of the Pleura, Peritoneum, and other Parts, which would not bear Depletion, and were cured by Calomel and Opium. By Mr. George Newstead.

We solicit the attention of our readers to the following statement. It will be read with interest, and probably with advantage, by those who unsheath the lancet the moment that pain is felt in abdomen or chest. There has been some peculiar constitution of the air, or rather of the earth, during the last few years, which has induced a host of anomalous diseases, imitating inflammatory affections, and leading to most injurious practice. Such practical facts as the following are very valuable.—*Ed.*

"A number of cases have occurred in my practice, during the last three or four years, which, with all the external characters of active inflammation, have not been relieved by bleeding, and, in fact, could not bear it to any great extent. The form chiefly assumed by the disease, when I first observed it, was that of pleuritis. Cold chills or shivering—uneasiness in the back and limbs, and frequently vomiting, were succeeded by very acute pain in the side. The tongue had the appearance exhibited in typhus mitior—the pulse was sometimes accelerated, but very often was not disturbed in the beginning—the secretion of urine was remarkably scanty, very high-coloured, and deposited a thick sediment. It sometimes terminated in three or four days with profuse sweats, and sometimes in a week or ten days by expectoration, tinged often with blood. The pain was so urgent, and the breathing so obstructed, that I did not hesitate to use the lancet; but the first bleeding generally put me upon my guard. I was astonished at the small quantity which commonly flowed before syncope was produced, and also at the slight relief of pain, even where larger abstractions could be borne. Cases like peritonitis began to occur, and I then found, that whether the patient complained of the chest or abdomen, the pain was not confined to one part. Upon examining those complaining of the chest, there was great tenderness to the *touch* there, (a circumstance I never remarked in inflammation of the lungs or pleura) and not only there, but upon the abdomen and very often down the back; and those who said the pain was in the abdomen were affected, in like manner, by pressure on the chest and back, as well as the belly. In some, even the arms and thighs were affected; and whatever part was touched, they shrunk like the subject of acute rheumatism upon handling an inflamed joint. This diffused pain upon pressure, and the diminished secretion of urine, I fixed upon as the characteristic symptoms of the disease. Although the region of the kidney was usually pointed out as the seat of the most acute pain in the abdominal disease, and the secretion of urine was so much disordered, there was not that frequency of making water, and pain in voiding small quantities, which mark nephritis. The state of the bowels was various—frequently diarrhœa came on with green stools, or a discharge of bloody mucus; but, as calomel was freely given, I attributed these symptoms to its use. One young man, however, before any medicine was given, had frequent discharges from the bowels of a thin bloody serum, without tenesmus, and totally different from any thing dysenteric. I observed some, where the chest was chiefly complained of, spit up the same kind of serum, like bloody water. The stomach was often irritable throughout the abdominal disorder, and a green fluid was occasionally discharged. I felt an awful responsibility at first, when I dared to treat this complaint without, or with very little, depletion; for patients themselves, identifying what they felt with what they had heard of inflammation, would ask to be bled, but I was alarmed by the exhaustion I had seen follow, and I never, except in two cases, ventured upon more than one bleeding, trusting afterwards to leeches, a dozen at a time. My reliance was upon opium and calomel, or mercurial frictions. I was partly encouraged to withhold the lancet by the state of the pulse, which was often not above 80 and natural to the feel, when the chest, back, and abdomen could not be touched without agony, and even the weight of the bed-clothes was irksome; for although I am aware that fatal inflammation of the bowels may exist without an accelerated pulse, I fancy that, commonly, it is when it proceeds from some mechanical obstruction, and that in pure enteritis or peritonitis, there must be a quick pulse, though the feel may be variable. The pulse did not often continue in this state—it generally got to be 100 or upwards after two or three days, when the febrile disorder, which seemed to me to modify and give a peculiar character to the inflammatory symptoms, had time to develop itself. My cautious practice has been successful. Out of a number of persons afflicted in this way, I cannot say how many, but I can readily bring forty to my memory, three died. Two of these had been freely bled, and the third was a woman seventy-eight years of age. Within the last month I have treated two cases successfully, even without leeches. I will give you a daily report of one of them.

"Jane Cotham, æt. 61. July 7, 1829. Attacked suddenly after tea this afternoon with excruciating pain all over the abdomen and vomiting. Eight o'clock, p. m. Complains of great pain in the abdomen, which is very much increased upon pressure — does not mention pain elsewhere; but, upon examination, the whole of the left side of the chest, as high as the axilla, and the back, are as tender to the touch as the abdomen—pain came on suddenly, but she has felt chilly and not very well all day —has been uneasy and stiff in her back and limbs two or three days—has never been subject to any spasmodic affection —pulse 72, with a sinking feel—tongue pretty natural—bowels moved both yesterday and to-day:—Warm bath—two grains of opium immediately. Pulv. ipecac. c. gr. x. Hydrarg. submur. gr. ij. cum dosi mist. salin. 4tis horis postea. Ol. ricini, ℥j. primo mane. Rub the parts affected, as much as can be borne, with camphorated oil.

"July 8th, 10 o'clock, a.m. Is easier. The pain on pressure continues, however, particularly acute on the left side of the chest, and the right side of the abdomen—cannot take a full inspiration—has no cough—urine said to be very little in quantity—no stool—has not yet taken the oil—pulse 72 without any sinking—tongue furred—ordered to take the oil and a black draught every four hours after until the bowels are opened. Eight o'clock, p.m. Opening medicine has not operated—does not complain much when she is still, but the whole of the abdomen is exquisitely tender to the touch, also both sides of the chest as high as the armpits—can bear pressure now upon the back—pulse 65—tongue a little moister —urine in very small quantity, but nothing particular in its appearance—has vomited after taking an opening draught. July 9th. Has been purged freely—does not complain of pain—can bear pressure tolerably well upon the abdomen, excepting the right side, which is still tender—has a little tenderness on the right side of the chest, but shrinks from the slightest touch on the left side—pulse 86—tongue loaded with a moist fur in the middle—evidently febrile action—has continued the calomel and comp. powder of ipecac. July 10th. Is easier—has slept well—bears pressure on the abdomen without pain, but it feels hard, and as if the muscles were spasmodically contracted—some soreness to the touch all over the chest. Pulse 80 — gentle diaphoresis — urine exceedingly scanty, depositing a thick sediment—tongue rather improving—dry and foul in the middle—bowels open —has vomited repeatedly.

"July 11th. Severe gripings — constant efforts to stool, but evacuates only small quantities of very bloody mucus— has passed, however, during the night, a large quantity of dark green feculent matter, mixed with scybala—no pain on pressing the abdomen—a little still on touching the left side of the chest—chalk mixture, with tinct. opii; three grains of opium for a suppository.

"July 12th. The griping and tenesmus abated after a dose or two of the mixture—returned this morning with some discharge of blood—used the suppository, and has been quite easy since—no pain on pressure—gums swelled and tender—pulse 100—urine still very scanty. Continue chalk mixture—to take ℥ss. ol. ricini in the morning.

"July 13th. Has had an easy night—castor oil has produced three good motions—mouth very sore—pulse 86—tongue beginning to clean—left off taking medicine.

"July 19th. Has been quite free from pain—bowels regular—fast regaining her former health.

"Two puerperal women have been severely attacked by the disease. One had two dozen leeches, and the other only one dozen very ineffective ones. Calomel and opium were given, and the bowels were opened once or twice with ol. ricini, combined with ol. terebinth. ʒij. Both recovered."

*Remarks.* It will hardly be contended that the above were cases of genuine inflammation. They were pleurodyne, and enteralgia, rather than pleuritis and enteritis. How far these complaints are allied to the aguish or periodical affections, now so very prevalent, may be a question. As Howden, in East Yorkshire, is situated near the banks of the Ouse, there is probably a source of malaria in that district, which may help to account for the causes of the diseases above described. We exhort our brethren to be strictly on their guard against excessive and indiscriminate depletion during the present reigning constitution.—*Ed.*

XXII.

